# Monitoring System Analysis for Evaluating a Building’s Envelope Energy Performance through Estimation of Its Heat Loss Coefficient

**DOI:** 10.3390/s18072360

**Published:** 2018-07-20

**Authors:** Catalina Giraldo-Soto, Aitor Erkoreka, Laurent Mora, Irati Uriarte, Luis Alfonso del Portillo

**Affiliations:** 1 ENEDI Research Group—Faculty of Engineering of Bilbao, University of Basque Country (UPV/EHU), Alameda Urquijo s/n, 48013 Bilbao, Spain; aitor.erkoreka@ehu.eus (A.E.); irati.uriarte@ehu.eus (I.U.); luis.delportillo@ehu.es (L.A.d.P.); 2 I2M—Institute of Mechanics and Engineering—University of Bordeaux CNRS (UMR 5295), site ENSAM, Esplanade des arts et métiers, 33400 Talence, France; laurent.mora@u-bordeaux.fr

**Keywords:** building energy monitoring system, Heat Loss Coefficient (HLC), fault sensor

## Abstract

The present article investigates the question of building energy monitoring systems used for data collection to estimate the Heat Loss Coefficient (HLC) with existing methods, in order to determine the Thermal Envelope Performance (TEP) of a building. The data requirements of HLC estimation methods are related to commonly used methods for fault detection, calibration, and supervision of energy monitoring systems in buildings. Based on an extended review of experimental tests to estimate the HLC undertaken since 1978, qualitative and quantitative analyses of the Monitoring and Controlling System (MCS) specifications have been carried out. The results show that no Fault Detection and Diagnosis (FDD) methods have been implemented in the reviewed literature. Furthermore, it was not possible to identify a trend of technology type used in sensors, hardware, software, and communication protocols, because a high percentage of the reviewed experimental tests do not specify the model, technical characteristics, or selection criteria of the implemented MCSs. Although most actual Building Automation Systems (BAS) may measure the required parameters, further research is still needed to ensure that these data are accurate enough to rigorously apply HLC estimation methods.

## 1. Introduction

In this paper, we introduce energy consumption in Europe, the role of the Heat Loss Coefficient (HLC) estimation in understanding the envelope effect on the Energy Performance of Buildings (EPB), the monitoring systems used for this estimation, and the role of fault detection in building energy monitoring systems. In Table A1, all acronyms used in this text can be found.

Butler and Dengel [1] define Heat Loss Coefficient (HLC) as the total heat loss from a building resulting from heat transfer through the envelope (walls, roof and floor) and from background ventilation per °C of temperature difference between inside and outside (expressed as W/K)). This review define the Thermal Envelope Performance (TEP) refers to a characteristic that can be used to evaluate the energy performance of a building envelope. The TEP can be characterized by estimation of the Heat Loss Coefficient (HLC), the energy consumption due to envelope performance or envelope’s characterization (*R*-value or *U*-value, dynamic thermal models of the envelope …).

Estimating the Heat Loss Coefficient (HLC) and the Thermal Envelope Performance (TEP) characterization of buildings are important to better understand their energy efficiency, in order to generate Energy Performance Certificates (EPCs). These can be employed as a tool to determine the discrepancies between performances at the design and operation phases of buildings. Actual TEP, actual energy equipment performance, and user behavior are the three main reasons for a building to consume energy differently than its design conditions suggest. European regulations ensure transparent and consistent EPCs through reliable methodologies to estimate the TEP of buildings. These methodologies used for HLC estimation in turn need to be fed with physical variables collected and processed by a Monitoring and Controlling System (MCS), which is composed of elements with the necessary precision to generate reliable EPCs. Likewise, MCS require faults to be detected and minimized in order to guarantee a higher level of accuracy in results by minimizing the error in calculations. Currently, existing smart buildings are monitored and controlled with various building systems (e.g., HVAC, heating, light systems), but do not have an integrated MCS of the type currently used in experimental tests to estimate the TEP and HLC.

### 1.1. Energy Consumption of Buildings in Europe

The potential for energy demand growth from connected devices in buildings, whether they are smart or not, has already been noted in many European Union (EU) markets, according to a study carried out by Statistics in the Control and Connectivity segment, ‘the number of active households is expected to amount to 43.7 m by 2022’ [2]. In the International Energy Agency (IEA) Central Scenario, 50% of household electricity demand for appliances by 2040 is expected to come from connected devices, presenting opportunities for a smart demand response, but also increasing the need for standby power. ‘Improving the operational efficiency of buildings by using real-time data could lower total energy consumption between 2017 and 2040 by as much as 10% compared with the Central Scenario, assuming limited rebound effects in consumer energy demand’ [3].

Increasing the energy efficiency of buildings can generate economic, social, and environmental benefits and improve the building performance, providing better comfort and wellbeing levels to users as health can be affected by indoor climate improvements. It is necessary to reduce the energy consumption of buildings, which represents approximately 40% of energy consumption in Europe, in order to reduce CO_2_ emissions [4].

Approximately 35% of buildings in the EU are over 50 years old. Of this percentage, only around 0.4–1.2% (depending on the country) are renewed annually. This implies that a greater renovation of existing buildings could generate significant energy savings through the reduction of 5–6% of the EU’s total energy consumption, and 5% of the total CO_2_ emissions [5]. The “Action Plan for Energy Efficiency: Realizing the Potential” calls to all regional and local authorities to develop energy efficiency plans and transpose into national legislation directives on the energy performance of buildings [6].

On 30 November 2016, the European Commission presented a proposal for a modest review of Directive 2010/31/EU on the Energy Efficiency of Buildings [7]. Some of the measures in the Clean Energy Package aim to meet the objectives of energy and climate of the EU 2030, together with the Directive on the Energy Efficiency of Buildings (EPBD) [8] which is aimed at achieving the EU’s energy efficiency objectives that are also addressed in the Energy Efficiency Directive (EED) [9]. The proposed revision of the EED (part of the Clean Energy Package) establishes a greater number of energy efficiency measures by 2030, in order for Member States to achieve at least 20% improvements in energy efficiency by 2020 [9,10]. This reduction of 20% is a matter of urgency in the action plan and is equivalent to around 390 Mtoe. This energy reduction is supported by the Green Paper on energy efficiency [11,12].

Energy efficiency and renewable energy technologies were the leading areas of Research, Development and Design (RD&D) investment of the European Commission in 2015, reaching significant shares (24% and 26%, respectively) of the total energy RD&D budget. RD&D for fossil fuels had the smallest share, accounting for 6% of the total budget in 2015 [13], and everything indicates that this investment trend will be maintained in order to carry out the measures in the Clean Energy Package and meet the 2030 goals.

### 1.2. The MCSs Role in Energy Performance Certificates and the HLC to Characterize the Thermal Envelope Performance of Buildings

The Energy Performance of Buildings Directive 2010/31/EU [7], in order to guarantee uniform conditions for its application, proposes to grant powers to the European Union for rating the smart readiness of buildings. These powers must be exercised in accordance with Regulation (EU) No 182/2011 of the European Parliament and of the Council [14]. This regulation specifies the need to have a smartness indicator that is used to measure the capacity of buildings to use Information and Communication Technologies and electronic systems, in order to optimize the operation of the building and to be able to interact with the network. The smartness indicator will create awareness among the owners of buildings and their occupants about the value that lies behind the automation of buildings and the electronic monitoring of technical building systems, increasing the confidence of the occupants in the ability to obtain a real savings when introducing new improved features in their dwellings.

In the same way, this directive talks about the importance of comparing EPCs issued before and after renewal. To do so, a transparent method provided by the installer of the certification or qualification level must be used to measure the performance of the equipment or material used for the renovation, thus guaranteeing their best use in the renovation of buildings in terms of the renovation quality, and to measure the associated financial impact and energy efficiency of buildings. To meet the objectives of the energy efficiency policy for buildings, the transparency of EPCs should be improved by ensuring that all necessary variables for calculations, for both certification and minimum energy performance requirements, are set out and applied consistently.

A report by the EPBD [15,16] remarks on the importance of implementing Monitoring and Control Systems (MCSs) to achieve quality assurance. These are an essential part of assessing compliance rates, which require confidence validation in source data and legitimacy in order to issue compliance reporting and can then be used for certification, such as the EPCs.

According to Article 3 of the EPBD [4], the EU Member must estimate a building’s energy performance using a specific methodology—at minimum using standardized conditions specified by national regulations. Taking into account the HLC is one of the Key Performance Indicators (KPIs) [17] of energy performance. 

To estimate the Heat Loss Coefficient (HLC) it is necessary to collect physical variable data of an in-use building or an unoccupied building, depending on the calculus methodology employed, to estimate the HLC. The sensors should measure, among other parameters, the temperature, heating, ventilating, solar radiation, and energy consumption [17,18] to demonstrate the energy efficiency of the building’s envelope. In-use building monitoring will be developed in the next few years to collect physical variables, and could be used to obtain the building’s envelope thermal characteristics.

The Thermal Envelope Performance (TEP) of a whole building is often quantified by the Heat Transfer Coefficient (HTC). ‘HTC’ is interchangeable with a second term, the heat loss coefficient (HLC), which has often been used when reporting Co-Heating results and will be used in this article to refer to the Heat Transfer Coefficient. ‘HTC’ has been adopted as a standard term in line with the naming convention used in ISO 52016-1:2017 [19], the international standard method for calculating the energy performance of a building, which cancels the previous standard ISO 13790:2008 [20]. The HLC is a useful metric that describes the total, time-averaged rate of heat transfer (in watts) from a building in a per-degree-Kelvin difference between indoor and outdoor air temperatures. Each building can be assumed to have a constant HLC—a value that is estimated as a metric in building energy models such as the Standard Assessment Procedure (SAP), which ‘is the United Kingdom (UK) government standard to calculate a buildings’ energy efficiency and carbon emissions’ [21]. By estimating the HLC, the thermal performance of the complete building envelope, as built, can be directly compared with the designed thermal performance independent of occupant behavior and weather conditions. 

### 1.3. The Monitoring Systems Used to Estimate HLC to Determine the Thermal Envelope Performance of Buildings

Given the importance of measuring buildings’ energy performance, this paper reviews the monitoring systems used to show the energy efficiency level of buildings through physical data collected by sensors. This data is used to estimate the energy performance using specific methodologies, which in future will guarantee transparent EPCs for minimum energy consumption. This article will focus on the energy monitoring used in projects to estimate the HLC with two methods: the Averaging Method [17] and the Regression Method, which is similar to the Co-Heating method [1,22]. These, along with other methods, can be used to calculate the Thermal Envelope Performance (TEP) of buildings. 

The latest report of Digitalization & Energy [3] of the International Energy Agency (IEA) in 2017 stated that there is a greater potential for energy saving in heating, cooling and lighting, since these together in 2015 accounted for more than 60% of the total demand for final energy in buildings. The report also highlights that sensors, intelligent controls, and connected devices consume energy to maintain connectivity, even when they are in standby mode. To improve the energy performance of the building this necessitates, for example, the use of intelligent thermostats to improve the management of heating and cooling loads, allowing an improved and even remote control of the temperatures throughout the building. 

Without automated monitoring and fault detection of the sensors and controls, performance can degrade. The number and range of types of sensors installed in commercial buildings is inadequate to provide sufficient automated (or even visual) monitoring [23].

The characterization of the TEP of in-use buildings and systems requires a monitoring system that provides real data, which in turn requires a minimum sensor set to obtain a correct characterization. The data collected from the sensor set then needs to be analyzed with different and robust methodologies due to the large amount of data obtained from the building monitoring systems.

Currently, some energy monitoring systems are integrated in domotic systems with the objective of giving information about the energy consumption and perform the control of user comfort parameters. However, in order to characterize the TEP of in-use buildings, there is no evidence of the integration of an energy MCS with a minimum sensor set in domoctic systems. This sensor set integer in Building Automation Systems (BAS) or domotic systems would allow us to know the real energy performance of buildings envelopes through TEP characterization after the construction or retrofit of buildings.

### 1.4. Fault Detection and Calibration in Building Monitoring Systems

Buildings may have operational problems due to degraded equipment, failed sensors, incorrect installation, poor maintenance, and improperly implemented controls. Currently, most problems related to building systems are detected through complaints from occupants or alarms provided by BAS. Detection and diagnosis can be performed automatically and integrally by integrating the experience required to detect and diagnose operational problems into software tools that take advantage of existing sensors and control systems. These tools are not designed to replace the people who operate the building systems, but to help them improve the functioning of those systems. The automatic start-up and diagnosis technologies for systems and building equipment are expected to reduce and act on problems and improve the functioning of the building, through the automatic and continuous detection of performance problems and maintenance requirements that are communicated to the building operators, who can then perform the necessary corrective actions [23]. 

Due to the large amount of data collected from sensor sets, is necessary to address which calibration system and methodologies are applied in the building energy monitoring systems, and to know the sensor set necessary to characterize the energy performance of in-use buildings’ envelopes. The literature related to fault detection and calibration in building monitoring is focused on the building systems, including fan coils, Heating, Ventilation and Air Conditioning systems (HVACs), heat pumps, air conditioners, commercial refrigerators, lighting, water heaters, chillers and cooling towers, Air Handling Units (AHUs), and Variable Air Volume (VAV) boxes. From all the works reviewed, a specific methodology that could be applied to the entire sensor of the BAS and domotic systems was not found.

## 2. Materials and Methods

**Figure 1:**
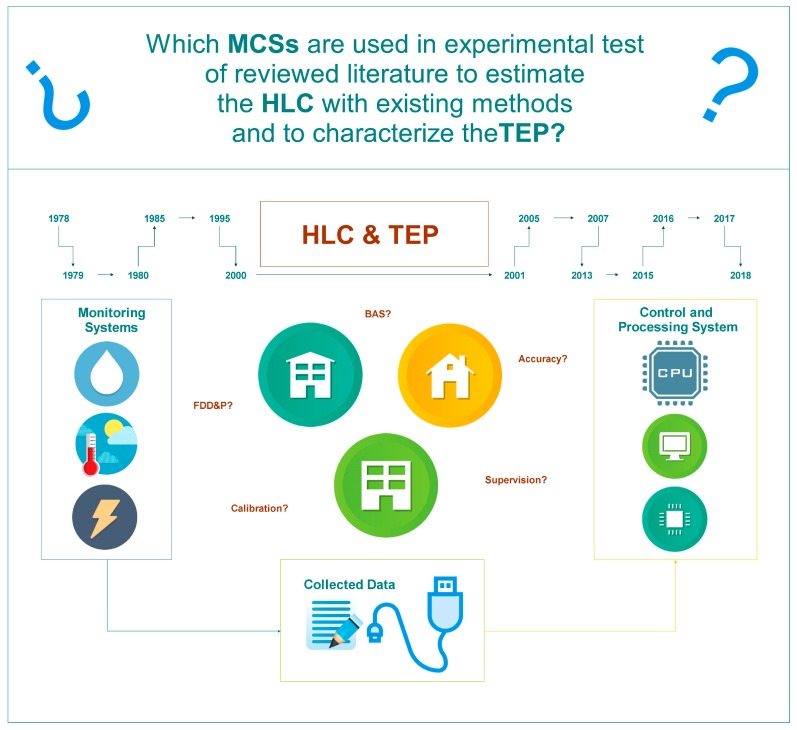
Abbreviated outline of Section 2: Materials and Methods.

This section begins with a review of building automation, communication protocols, sensors and the fault detection methods most used in building control systems. This gives a perspective on the monitoring and controlling of the system that is necessary in building automation. In order to identify and analyze the MCSs implemented in current research projects, we undertook a review of the literature that, through experimental tests, estimated the HLC using the Average Method, Co-Heating Method, and other methods to characterize the TEP. This reviewed literature allowed the identification of the equipment that makes up MCSs, and that is used to collect and process the physical variables in these experimental tests. Figure 1 shows an abbreviated outline of the development of this section.

### 2.1. Building Automation

The Building Control System, also termed the Building Automation System (BAS) or Building Management System (BMS), is the control system composed of integrated hardware and software networks that monitors and controls the indoor climatic conditions in building facilities [24]. 

The Building Automation System (BAS) is installed to monitor and control the heating, cooling, ventilation, air conditioning, lighting, shading, life safety, alarm security systems, and other building systems [25]. The system can be divided into four areas: applications, hardware, communications, and oversights [23]. The BAS is a part of the Intelligent Building, where this ‘intelligence’ implies capturing the current state of the building and its devices through the collection of physical variables and signal processing to make the appropriate adjustments, so that the building inhabitants experience increased marginal utility in terms of comfort and energy cost. Intelligent buildings increase this marginal utility through sensor system integration, computer automation, information and communication systems, smart home appliance devices, and new materials [26]. 

‘Domotic’ is other term used frequently in reference to building automation; it is defined by S. Millán-Anglés [27] as a scalable set of services integrated into the home that are provided by systems that can configure one or several internal networks of the habitat and that, in turn, can communicate with networks outside the home. These services have functions related to energy saving, technical management of facilities, information, communication, leisure, accessibility, assistance, comfort, and more.

Georgios Lilis [28] defined three hierarchical level of functionality in a BAS. The management level is where all information is collected, aggregated, and represented for further management by the operator. The automation level includes the entire infrastructure for controlling and applying management of the data or system supervision, in which interacting devices range from environmental sensors for luminosity, humidity, temperature, presence, and so forth, to actuators controlling passive devices and environmental parameters such as heating, lighting, and access to premises. Finally, the field level is where all the end-devices and field buses which interface the physical world and are used in the automation of industrial processes and buildings, and which are limited solely to point-to-point communication within the BAS, belong.

The functions of a Building Automation and Control System (BACS) generally include the Heating, Ventilation and Air Conditioning systems (HVACs); domestic hot water; lighting system control; shading systems control; energy conversion and storage (heating and cooling); onsite power generation; monitoring and data acquisition; and communications and security management [29]. Building automation integrates technology in a closed space with intelligent designs, which in turn can be integrated by indoor and outdoor communication networks—wired or wireless—so that energy management is efficient and includes the air conditioning and boiler controls, awning controls, and electric shutter and electricity management.

Economic and legal restrictions regarding energy consumption and environmentalism define building energy borders [26]. House system optimization is possible through the Control System of the BAS, which helps to improve the comfort of the occupants while reducing the energy consumption and expediting the operation, monitoring, and maintenance of the building [25]. The reduction of electricity consumption and improvement in the occupant comfort level make the building an energetically efficient system, which is largely achieved by the interaction of a wide range of sensors that collect physical variables, such the temperature, CO_2_ concentration, zone airflow, daylight levels, occupancy levels, and so forth [23]. Even so, energy management is conditioned by user behavior and comfort conditions have to take into account the lighting control and heating and cooling system control in the building automation.

### 2.2. Protocol Communication Used in Building Automation

Communications play a major role in enabling building-wide controls. The communication protocols let communication between devices occur, and are central to data transmission in order to share essential information that allows effective control functioning. This transmission uses physical media through which control information and commands pass between devices via twisted-pair wiring or wireless devices, and has a substantial impact on the installed cost of building controls in building automation systems [23]. Table 1 shows various analogies between the wired and wireless communication protocols.

Today, building automation systems can be realized using a multitude of different standards. In the 2010 IEEE International Symposium on Industrial Electronics, the main building automation protocols were identified as [30]:

1. KNX is an international standard (ISO/IEC 14543-3), European (CENELEC EN 50090 and CEN EN 13321-1) and Chinese (GB/T 20965), open for control in both commercial and residential buildings [31];
2. LonWorks standard is based on the scheme proposed by LON (Local Operating Network). The standard has been ratified by the American National Standards Institute (ANSI) organization as official in 1999 (ANSI/EIA 709.1-A-1999 [32];
3. BACnet is a Data Communication Protocol for Building Automation and Control Networks. Developed under the auspices of the American Society of Heating, Refrigerating and Air-Conditioning Engineers (ASHRAE) 13 5-1995-7 and published in 1995, the BACnet standard has the objective of providing a solution to the systems of automation and control of buildings of different sizes and types [33];
4. EnOcean is the standard based on the Institute of Electrical and Electronics Engineers (IEEE) 802.15.4. Where the modules based on EnOcean technology combine micro power converters with very low power electronics. This technology allows wireless communication between wireless sensors without batteries, switches, controllers and gateways. EnOcean is a wireless energy capture technology used in building automation systems and other industrial applications, transportation, logistics and smart homes [34];
5. Zigbee specifies a set of high-level wireless communication protocols with low-power digital transmission, based on the IEEE 802.15.4 standard for Wireless Personal Area Networks (WPAN) [35].

Currently, the BACnet, LonWorks, KNX and ZigBee technologies (based on IEEE 802.15.4) have attained considerable weight in the global market, as KNX has a strong presence in the European market [28]. Other technologies frequently used in BAS are:

1. INSTEON is a domotic network technology designed by SmartLabs, Inc. (Irvine, CA, USA). It is designed to allow devices such as switches, thermostats, sensors (movement, heat, smoke etc.) to be connected in a network through the power line and the radio frequency [36];
2. Modbus is a communications protocol located at level 7 of the Open System Interconnection (OSI) Model, based on the master/slave architecture (Remote Terminal Unit), or client/server (Transmission Control Protocol/Internet Protocol (TCP/IP)), designed in 1979 by Modicon for its range of Programmable Logic Controllers (PLCs). Developed into a de facto standard communications protocol in the industry, it has the greatest availability for the connection of industrial electronic devices [37];
3. Z-Wave is a wireless communications protocol used mainly for home automation. It is a mesh network that uses low-energy radio waves to communicate from one device to another, allowing wireless control of appliances and other devices [38].

The low-power wireless communication protocols such as EnOcean and Z-Wave are generally used in home automation and industry. Similarly, INSTEON is not restricted and gives support for wireless communication, and while it is generally used for home automation it is not limited to this [25]. According to a report from the Superior Council of Scientific Investigations of Spain (CSIC report (2014)) [27], the most used communication protocols are Wifi, Ethernet, and Bluetooth. As for the control protocols, these are the European Installation Bus and KNX. 

On the other hand, the most used framework platforms are Lonworks, Universal Plug and Play (UPnP) architecture; which is an open architecture and allows the interconnection between devices such as personal computers, home appliances, consumer electronics devices and wireless devices [39].

Moreover, Open Services Gateway Initiative (OSGi) that began in 1999 as a set of standards for a Java-based service framework that could be managed remotely. OSGi was originally conceived as a gateway to manage smart devices and other Internet-enabled devices in the home [40].

To translate the protocol information used in an initial network, to the protocol used in the destination network, gateways are used. The approach based on gateway has several disadvantages, storage of large mapping tables is required, and this is a factor that limits the scalability of the BAS since the effort required for configuration and maintenance increases with the translation of all the relevant data points that are incorporated from the appropriate segments. This is a significantly large mapping table to be stored and can be a limiting factor with respect to BAS scalability. In addition, having a front door can introduce a single point of failure and a security risk [41]. The literature demonstrates designs for multi-protocol devices, since this is a gateway-free solution that eliminates the need for specialized gateways for inter-protocol communication, increasing the potential product range available for each manufacturer and decreasing the installation cost and number of devices needed for building automation [41]. 

Unfortunately, protocols used in building automation are often not compatible with each other, therefore inter-operation across system boundaries requires special gateway solutions. To counteract these limitations, several middleware solutions have been developed that allow the communication of adjacent sides so that there is abstraction of the specific details of the provider of the BAS components [42]. This solution (Middleware) is a software that allows interaction and communication between various applications or packages of programs, networks, hardware, and/or operating systems. The communication hidden the resources heterogeneities of software, operating system, protocols, and so forth determine the interoperability between them [43].

Currently, there is no intrusion detection and prevention available for the BAS networks, which are increasingly extending their functionalities and their connection to internet. This significantly increases the exposure of BAS networks to cyber-attacks due to the significant increase in the attack surface. This also increases the interconnection between communication protocols due to the increase in information services and advanced network technologies, with the need for Cloud Computing and Fog Computing increasing in order to provide solutions for the automation of final physical devices [44], allowing its integration on Internet through a virtual representation, being the vision of Internet of Things (IoT) [45]. The building automation devices are considered for an integration in the IoT in order to have a smart and sustainable building operation [46]. Besides, the main difference between and Fog computing is the Cloud computing ‘refers to both the applications delivered as services over the Internet and the hardware and systems software in the data centers that provide those services’ [46], and Fog computing is a ‘paradigm that extends Cloud computing and services to the edge of the network. Similar to Cloud, Fog provides data, compute, storage, and application services to end-users’ [47].

### 2.3. Sensors Used in Building Automation

The sensor systems in advanced intelligent buildings are required to provide comfort, high performance and automation, energy and resource savings, and security [26]. In 2010, many modern automated buildings contained a limited number of wired sensors in control systems such as BACnet or LonWorks. This is mainly because the wired sensors need additional wiring for each sensor, wich is a significant barrier in wired sensor deployment due to the increased installation cost. The entrance into the market of low-cost wireless sensors without a need for wire has opened opportunities in the market to increase the number of connected sensors in buildings, thus allowing for improved sensing of the different necessary variables to achieve efficient and effective automation and consequently improve user comfort [48].

For the correct control of the interior conditions, a considerable number of sensors is necessary to control unwanted levels by the users and to achieve optimum levels in the use of energy. It is also necessary to use optimal control techniques in the system and throughout the building to achieve the levels of performance necessary to ensure that the conditions inside the building are of high quality with a minimum consumption of net energy [23,25,26,49].

Previous research has specified the use of sensors and meters for controlling building performance, where the most installed environmental sensors are those measuring temperature, Relative Cumidity (RH), and Carbon Dioxide (CO_2_)—which are used to control the HVAC operation. This control through environmental variables looks to maximize user comfort with an optimal performance of the HVAC systems [29]. The sensor used to meter electrical power/current is one of the most important types of sensor employed for monitoring energy efficiency. Table 2 shows a list of the main sensors and meters used for control in building automation. 

In the literature, there is no specification of the monitoring system necessary to estimate the energy performance of a building’s envelope using a specific methodology, according to Article 3 of the EPBD [4]. However, there are many studies that have been carried out to estimate the HLC in order to characterize the building’s thermal envelope performance, together with other estimations to characterize the energetic behavior of the building envelope. The monitoring systems needed to measure the user’s behavior and comfort are studied in depth in many papers through the control of heating, cooling, and lighting systems, measuring the electrical consumption of homes and buildings in order to know the energy performance of the users. In the next section, we will present the monitoring system necessary to estimate the HLC using Average Methods [17] and the Co-Heating Method or similar [21,22] and will present a review of different monitoring systems used in different studies developed to estimate the HLC.

### 2.4. Fault Detection, Diagnostics, Pronostics and Calibration in Building Monitoring Systems

Evaluating uncertainties in a test can lead to comprehension errors due to the absence of knowledge about the “true” value of a measured variable, especially systematic errors due to the absence of a reference between the true value and the measured value. The true value of a measurement can never be known, but when you measure the HLC of a building it varies in an unknown way and is difficult to predict, and this makes it difficult to assess the uncertainty of the estimate [50]. Some authors have studied the uncertainty in the calculation of the HLC, such as Stamp S. [51] who investigated the uncertainties related to solar gains through field tests and simulated Co-Heating tests.

Sensor errors greatly affect the performance of control, diagnosis, and optimization systems within building energy systems, negatively affecting energy efficiency. Calibrated measurements improve the accuracy of energy performance analysis for a building energy system by up to 18% [52]. It has been reported that the exponential increase of the number of maintenance requests for building energy systems in the past decades is due to an increase in building operational faults [53]. Typical operational faults may arise from improper installation, equipment degradation, sensor offset or failures, or control logic problems. The latter can be split into several categories: control faults, sensor offset, equipment performance degradation, fouling faults, stuck faults, and others [54]. 

Table 3 shows impact sensor errors in a monitoring system, which reflect the need to implement and integer a tool to detect, predict, diagnose and calibrate the sensor and monitoring systems in all building automation systems in an integral way. 

Automated Fault Detection and Diagnosis (AFDD) is an area of investigation concerned with automating the processes of detecting faults [60], whereby faulty operations, degraded performance, and broken components in a physical system are detected and understood. AFDD tools are based on algorithms that process data to determine if the source of the data is experiencing an error. The tool can be passive if the operation of the equipment/system is analyzed without modifying any reference points or control outputs, or active if the changes are made automatically to produce or simulate the operating conditions of a wider range of conditions that could not be modified for some time in a normal operation [23]. The impact of the failures allows determination of the priority of repairs, directly affecting the reduction of energy use and costs and achieving greater comfort and useful life of the equipment, as well as a reduction in service costs. The severity of the failure and its impact on energy consumption is essential to consider in order to prioritize repairs. Assessing the failure or evaluating the impact (energy and cost) is one of the main steps in the AFDD process, however determining the severity of the failure is difficult because in many cases the information necessary to perform the evaluation is not readily available [60].

The sensor and control performance can degrade without automated monitoring and fault detection. The number and range of the types of sensors installed in buildings today is inadequate to provide sufficient automated (or even visual) monitoring. Performance monitoring, automated fault detection and diagnosis, commissioning, optimal control, and the use of developed environments, design tools, and trainers are complementary technologies, with notable potential to realize significant energy savings and other performance improvements in commercial buildings, including existing buildings [23]. All sensor systems are facing a noticeable upward trend in performance requirements for maintenance, downtime, reliability, fault tolerance, fault recovery, and adaptability [26]. 

The main fault detection and calibration methodologies in building systems include fan coils, HVACs, heat pumps, air conditioners, commercial refrigerators, lighting, water heaters, chillers and cooling towers, AHUs, and VAV boxes [60].

**Figure 2:**
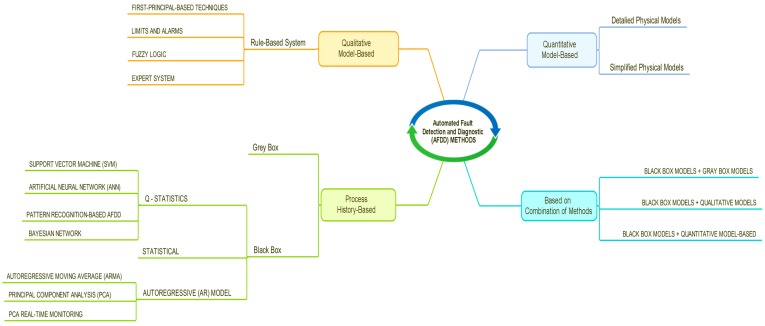
Classification scheme for AFDD methods based on previous research [60,61].

The AFDD methods can be classified into quantitative model-based, qualitative model-based, and process history-based methods [60,61,62] (Figure 2). The history-based process is the most used when the theoretical model of system behavior is inappropriate to explain its behavior, or it is not easy to create the model. In this AFDD method the Black Box is the most used because of its simplicity. The qualitative model-based (rule-based) method is the second most used AFDD method. The quantitative model-based method needs a precise mathematical model of system behavior and reliable sensors for the acquisition of data—as it is the most complex model and the least popular, it is more used for industrial purposes than building landscapes. There are also AFDD methods that combine these three methods, which are used in order to improve the efficiency of individual methods and detect failures simultaneously (e.g., rule-based combined with statistical methods to reduce the noise, disturbances, and uncertainty of monitoring) [60].

AFDD can be integrated into an automatic start-up process. Start-up (new buildings) and commissioning (existing buildings) involve functional tests carried out to determine if a device or system is working correctly. In the commissioning process, the proper functioning of the equipment is verified by observing a series of functional tests, however it is not guaranteed that the equipment can continue to function properly. Only continuous monitoring of the state of the equipment and its performance can guarantee continuous operation. The AFDD system constantly monitors the equipment and identifies failures and loss of performance, and is a fundamental system in the commissioning of buildings. While the intervention of a human operator or repair technician is essential to complete the start-up cycle, without the automated monitoring system operating continuously many problems may not be detected for days, weeks, months, or even years [23].

All studies on fault detection and calibration of monitoring systems that were reviewed did not apply AFDD methodologies in the energy monitoring system to characterize the TEP and to understand the energy efficiency of in-use buildings’ envelopes through HLC estimation. Currently, the methods lack a holistic approach to predict the global impacts of faults at the building level [60].

### 2.5. Monitoring Systems to Estimate the Heat Loss Coefficient (HLC) Using the Average Method and Co-Heating Method

To guarantee transparent EPCs for minimum energy performances, it is necessary to estimate the energy performance using specific methodologies in order to determine the energy efficiency level of buildings. Physical data collected from the sensors of a monitoring system are necessary for this purpose. This section will focus on the study of energy monitoring systems used in projects to estimate the Heat Loss Coefficient (HLC) with two methods: the Averaging Method [17] and the Co-Heating Method, or similar [1,22].

#### 2.5.1. Methods and Data Requirements to Estimate the Building Envelope HLC

The monitoring requirements of the Average Method and Co-Heating Method were analyzed, showing the main physical variables required and reviewing the monitoring used in different projects published to estimate the HLC.

Corrected Average Method: A. Erkoreka [17] proposed the Average Method and Corrected Average Method, with similarities to the ISO 9869 standard, to estimate the Heat Loss Coefficient (HLC) of the whole building. These methods take into account the *k* observation of all heat gains inside the building (including the heating system and all the other internal gains, but excluding solar radiation) represented by (*Q* + *K*) and the solar gains (*S_a_H_sol_*) (Equation (1)) in specific periods where:

- There is very low solar radiation and it is possible to roughly estimate the building’s solar heat gains. To minimize the uncertainty of roughly estimating the solar gains, the solar gains should be less than 10% compared to the sum of all the rest of the heat gains inside the building (*Q* + *K*).
- The interior to exterior average temperature difference during the selected testing period should be higher than 15 °C and never less than 10 °C. Furthermore, the building’s average temperature must be the same at the start and end times of the method to make the effect of the change in internal energy of the building negligible.

(1) $$\;HLC_{N,air-to-air}=\;\frac{\sum_{k=1}^{N}\left(Q_{k}+K_{k}+S_{a}H_{sol,k}\right)}{\sum_{k=1}^{N}\left(T_{i,k}-T_{o,k}\right)}\;$$

where:

*HLC_N, air-to-air_* [kW/°C] is the air to air Heat Loss Coefficient of the building envelope. The HLC considers both, losses due to transmission and losses due to ventilation and/or infiltration.

*Q* [kW] is all the heating and ventilating systems’ energy inputs inside the building.

*K* [kW] is all the other heat gains inside the building (illumination, all other electrical device consumption, and heat gains due to people, solar gains, and *Q* are not included).

*T_i_* [°C] is the indoor air temperature.

*T_o_* [°C] is the outdoor air temperature.

*S_a_* [m^2^] is the solar aperture.

*H_sol_* [W/m^2^] is the horizontal global solar radiation.

*k* is the index observations for the period consisting of *N* measurements of all variables.

To solve this estimation, the physical variables are obtained from five different types of sensors, shown in Table 4. 

Co-Heating Method: The Co-Heating test [1] has existed for more than three decades and been used for many purposes. The performance parameters of the building of interest, in the form of the Heat Loss Coefficient (HLC) and the global solar aperture, are determined by applying a linear regression analysis, assuming a simplified thermal equilibrium and aggregate performance data. Therefore, we observe the aggregate performance of its components. A common method to evaluate this is the Co-Heating test. This test essentially represents an almost stationary test based on the linear regression analysis of the aggregate building performance data acquired during the appropriate heating experiments. During a Co-Heating test, the investigated dwelling is heated homogeneously to an indoor temperature of a steady state of 25 °C, using electric heaters and fans scattered throughout the building. The use of electrical energy, indoor and outdoor air temperatures and relative humidity, wind speed and direction, and solar radiation are controlled throughout the test. The influence of the transient effects induced by the loading and unloading of the thermal mass of the building can be reduced by carefully selecting the period of the experiment and averaging the collected data over a sufficient period.

Using the regression analysis, the indoor and outdoor supervised conditions are related to the electric heating energy necessary to maintain a constant indoor air temperature. The coefficient that describes this relationship, representing the thermal performance characteristics of interest, is the Heat Loss Coefficient (HLC) in W/K. The total HLC constitutes a combined loss due to heat transmission and infiltration/ventilation. To decouple both, a Co-Heating test is usually combined with a blower door test or tracer gas test [1,18,22,63,64].

According to the specifications of the standard ISO 13790 [20], it is possible to obtain measurements to estimate the HLC of a dwelling through a Co-Heating test, determining the heat loss throughout the building envelope. The heat loss of the building achieved by the Co-Heating test has some advantages over other possible estimates of independent mechanisms of heat loss; for example, infiltration measurements [65] or point measurements (e.g., measurements of the building envelope independent component *U*-values, in situ [66]).

D. Butler (2013) [1] used the regression methodology to estimate the HLC (Equation (2)) and the solar aperture (*S_a_*) of the whole building with reference to the south vertical global solar radiation. The Co-Heating test is carried out in winter to reduce the uncertainty effect of solar radiation on the HLC.

(2) $$\;\left(Q+K\right)=\;HLC\left(\Delta T\right)-S_{a}V_{sol}\;$$

where:

*Q* [kW] is all heating and ventilating systems energy inputs inside the building.

*K* [kW] is all the other heat gains inside the building (solar gains and *Q* not included).

∆*T* [°C] is the difference between *T_i_* [°C] (the indoor air temperature) and *T_o_* [°C] (the outdoor air temperature).

*S_a_* [m^2^] is the solar aperture of the whole building with reference to the south vertical global solar radiation.

*V_sol_* [kW/m^2^] is the vertical south global solar radiation.

To solve this estimation, the physical variables are obtained from five different types of sensors, shown in Table 5.

#### 2.5.2. Sensor Accuracy of Monitoring Systems Used in an Experimental Test for Evaluating the Building Envelope HLC: A Research Project Sample

In order to have a reference and know the current accuracy used in the experimental test to estimate the HLC, this section presents the sensors used in an occupied big office building (Table 6) together with the communication protocol, hardware, and software (Table 7) that was implemented. The MCS was implemented in a public building of the University of the Basque Country under the 7th Framework Program for Research (FP7) [67] project A2PBEER [68,69] in which an energy characterization [17] was carried out. This building has been retrofitted and is currently being energetically monitored. Their MCS had not implemented any FDD methods.

This project is a sample of how the automation of buildings is being implemented in research. This example and the literature studied in the next section demonstrate the need to implement MCSs in experimental tests to obtain the energy characterization of the building envelope and a correct estimate of the HCL.

#### 2.5.3. Equipment of Monitoring and Control Systems (MCSs) Used in Research Projects to Estimate the HLC and Characterize the TEP of Buildings: A Review of MCSs in Experimental Tests

In order to know the MCSs used to measure the physical variables necessary to estimate the HLC and TEP, a range of literature has been selected. The purpose of this selection is to identify sensors, controls, hardware and software employed in research studies in order to determine, for example, what kind of accuracy and technical sheet the used sensors have. In addition, the technology used will be analyzed in the discussion section to understand the possibility of implementing the MCSs used in BAS and domotic systems in order to characterize TEP.

The choice of literature took into account several requirements in order to ensure the literature was based not only on an analytical study of HLC estimation and TEP characterization, but also had an experimental basis. The experimental basis should be specific to buildings, housing or prototypes. Within the selection, studies based on simulations or that are purely theoretical or analytical were not taken into account. The requirements for the research to be considered were:

(1) Studies based on experimental tests of buildings, houses, or prototypes of small scale.
(2) Studies that were developed with the objective of characterizing TEP in experimental buildings, houses or prototypes of small scale, and that also used one or more of following methods:
  (a) Co-Heating Method.
  (b) Energy Balance.
  (c) Average Method.
  (d) Corrected Average Method.
  (e) Other methods (e.g., statistical methods) for estimating the building envelope energy behavior, but that also include at least one of the following studies:
    - Energy Consumption.
    - Energy Balance.
    - Infiltration.
    - Local *U*-Value.
    - Other energy analysis (e.g., estimation of the heat dynamic of buildings).

Table 8 shows the relationship of the references selected for this study with the corresponding methods and studies carried out from 1978–2018. The references include reports, journal articles, and conference publications. The literature that was studied includes reports of the first studies of the Co-Heating Method in the 1970s [70], developed by the U.S. Energy Department [71], which analyzed the sensors, controls, instrumentation, hardware and software necessary for MCSs to achieve HLC estimation and other building’s envelope energy behavior estimation to characterize the TEP of buildings. Moreover, in the second decade of the 21st century, an increase of experimental tests was observed, with a greater concentration of publications occurring in 2015, 2016, and 2017. A study from 2018 [72] exists in which a sensitive analysis was carried out to determine the level of uncertainty in the HLC estimation due to the measurements obtained by the sensors. This type of analysis is necessary in order to identify which type of sensors should be implemented in the MCSs of buildings in order to characterize their TEP.

Table 9 shows the sensors, controls, hardware, software, and devices used in the experimental tests of each selected reference, together with the verification of the FDD method that was used, which was not implemented in any of the experimental tests studied. In the next section the results and analysis of MCSs are developed through qualitative and quantitative analyses. Additionally, the methodology and criteria used to obtain the results are described.

## 3. Results and Discussion

This section will present the qualitative and quantitative analysis of the equipment and the technical specification level of the MCSs utilized in 24 bibliographies that were reviewed, of which 67% used the Co-Heating Method and 17% used other regression methods. One publication estimated the HLC with the Corrected Average Method and another with the Average Method, with each one representing 4% of total publications reviewed. To estimate local U-Values, two of four publications used ISO9869:1994, one publication does not specify the methology implemented, and another was based on ISO 6946:2007 [85]. Seven publications, or 29%, implemented other methodologies to characterize the TEP—for example, statistical methods. These values are specified in Tables 13–15.

The objective of the qualitative and quantitative analyses is to identify the MCSs currently used to estimate the HLC and TEP in order to:

- Identify the technology used in experimental tests.
- Analyze the integration of MCSs into BAS.
- Identify the currently state of FDD methods implemented in MCSs.

Based on data recompilation of Table 9, a qualitative and quantitative analysis were undertaken in terms of the function of the equipment and technical specification level of the MCSs’ described and presented in the methodology reviewed from the literature. 

To analyze the MCS technologies used in experimental tests of the selected bibliography; different levels have been defined according to technical specifications that selected publications describe in their experimental methodology. For this, the MCS equipment implemented to collect and process the physical variables to develop the methods for the TEP characterization, which these publications propose, is characterized. The three levels are defined as Level A, B, and C. These levels are quantified as 1, 0.5 and 0, respectively, and the degree of detail that defines the level is shown in Table 10.

The evaluated criteria has been divided into two groups: one to analyze the Monitoring System that include the sensors, and other to analyze the Controlling System that include controls, communication protocols, software and hardware. Table 11 shows the criteria considered to analyze the MCSs’ specification of the degree of technologies used in research projects in the reviewed literature. The MCSs’ specification degree helps to identify the degree of importance of MCSs in HLC estimation and in other estimates used to determinate energy behavior of the buildings, in order to characterize the TEP. This allow us know the reason there is a difficulty in identifying MCS technologies used in experimental tests.

Table 12 shows the review bibliography with the analyzed criteria and the corresponding level for each of them. In analyzing the Monitoring System device criteria, more than 50% of the literature studied falls into level C—where it has not been possible to identify the type or model of the sensors used in those experimental tests. Of 24 bibliographies reviewed an 83.3% did not specify the data sheet, and 58.3% did not specify the sensor’s accuracy either. A total of 79.2% did not describe the decision criteria used to select the sensors (economic, technical, or other criteria). Furthermore, a 67% used the Co-Heating Method and 17% used other regression methods. One publication estimated the HLC with the Corrected Average Method and another with the Average Method, with each one representing 4% of total publications reviewed. To estimate local U-Values, two of four publications used ISO9869:1994, one publication does not specify the methology implemented, and another was based on ISO 6946:2007 [85]. Seven publications, or 29%, implemented other methodologies to characterize the TEP—for example, statistical methods. These values are specified in Table 13, Table 14 and Table 15.

Regarding the Controlling System devices, they showed a similar tendency as the Monitoring System devices, with only around 12.5% giving a complete description specifying the model or type of control devices, the data sheet, and the criteria used to determinate the Control System in terms of the function of its technical requirements. On the other hand, 20.8% specified the protocol communication, software, and hardware used. In addition, just 25% specified the operating characteristics of the hardware and software used to control and process the collected data, respectively, around 21% and 13% specify the hardware and software type and the criterion used to determinate the controlling system implemented.

By studying the publications, it was often possible determine the sensors and devices used when these were not included explicitly in the methodologies because they were specified in the analysis, tables, and/or data graphics. In this way, it was possible to know in some cases the sensors used in the experimental test. Even so, there are publications that did not specify the devices used and just gave the results, making it impossible to identify the devices used in the experimental test. Table 14 and Table 15 show the sensors, devices, software and hardware identified in the selected literature. It was possible to identify in 100% of literature the use of sensors to measure the interior temperature, in 83% those used to measure the exterior temperature, and in around 13% those used to measure surface temperatures. The difference between the exterior and interior temperature may be because these data were collected using weather stations, but neither was found if this measure was collected by a station. In the tests, just 13% and 17 had used sensors to measure indoor CO_2_ level and indoor relative humidity respectively.

In 21% of experimental tests, infrared thermographics were used, and 50% used different devices to estimate the infiltration. Only 33% used local heat flow sensors. The environmental conditions were measured in several tests, of which 50% specified the horizontal global radiation, 63% the vertical global radiation, and 33% the diffuse radiation and relative humidity sensors.

In 83% of the reviewed literature, an electricity meter was used to measure the total energy consumption. Likewise, in determining the use of other meter sensors, 17% used gas meters, 25% used heat meters, and 8% used HVAC air flow and specific sensors to measure the light electricity consumption.

A total of 67% used the Co-Heating Method for their experimental tests, whereas 94% specify the use of a meter to measure the total electricity consumption and 81% the use of a sensor to measure the exterior temperature; of these, 25% measured the exterior RH and only 13% measured the interior RH. Respectively 50% and 38% measured the global vertical and global horizontal solar radiation, as well a 19% wind speed and 25% measured diffuse solar radiation and wind direction, just 56% and 50% described the use of electrical radiators and fans, respectively. A total of 56% measured air infiltration, 31% used infrared thermographics, 38% used heat flow, and only 6% measured surface temperatures. A 25% and 19% used heating and HVAC systems severally, the use of these building systems in some cases was to maintain the external conditions when a building prototype was being tested, or to avoid stratification during different tests. The physical variables shown in Table 5 are those measured in the Co-Heating Methods developed in selected publications.

Experimental tests in 75% of the reviewed cases that used other regression methods used sensors to measure the total electricity consumption, outdoor temperature, exterior relative humidity, vertical solar radiation, and wind direction, and 50% measured the surface temperature, indoor CO_2_ concentration, heat flow, gas consumption, and diffuse solar radiation. All of the reviewed research experiments used sensors to measure global horizontal radiation, wind speed, and heating meters. Another 25% with sensors measured the interior relative humidity, infiltration, interior illumination level, light electricity consumption, and exterior illuminance.

The Average Method used in experimental test, specified devices to measure the interior, exterior and surface temperature, heat flow, electricity consumption, exterior relative humidity, horizontal, vertical and diffuse solar radiation, direction and wind speed, besides the use of heating meters. The Corrected Average Method specified measures of the interior and exterior temperature, indoor CO_2_ concentration, interior and exterior RH and light power consumption, indoor and outdoor illumination levels and horizontal solar radiation, wind speed and also uses heating meters. Each of these methods were used by only one of the experimental tests.

Seven papers (29%) used methods different to Co-Heating, regressions, the Average Method, and the Corrected Average Method for TEP characterization. Two publications used devices to measure the surface temperature, heat flow, indoor CO_2_ concentration, and gas consumption. Six experimental tests did not measure the interior RH, interior illuminance level, and HVAC airflow. A total of 71% used a sensor meter to measure the total electricity consumption and measure the global horizontal solar radiation, 86% measured the outdoor temperature, and 100% measured the global vertical solar radiation. A total of 57% measured the diffuse radiation, and 43% the exterior RH. Only one publication used an electrical radiator or a heating system, while in two publications an HVAC system was used. A 43% used heat meters and measure infiltrations versus a 57% that measure the direction and wind speed.

Analyzing the devices of Controlling Systems of all literatures, around 42% used a thermostat and 25% used any other device for another purpose, for example to open or close windows. Around 56% of publications that analyzed the Co-Heating test used thermostats, versus 14% of publications that implemented other methods to estimate the energy performance of buildings envelope. Besides the 50% used fans and 56% electric radiators, versus 33% and 42% respectively of all literatures.

No project mentions the use of a Supervisory Control and Data Acquisition (SCADA) for collecting and processing data, although around 54% used a data logger and approximately 33% a data processor.

The communication protocols that were used were not identified; only around 21% of publications specified some characteristic of data transmission, and only one publication specified the use of a gateway or transmitter. A total of 29% of all publications specified the use of computer. The experimental tests that implemented the Co-Heating and other methods to estimate the energy behavior of buildings envelope to characterize TEP, have the same tendency.

The results show that no publication has implemented an FDD method to detect, identify, and correct the error in MCSs used during experimental tests.

## 4. Conclusions

There is evidence that energy efficiency research is primarily focused on the use of automated projects to collect physical data, transfer the information using standard communication protocols, and through the use of software process all information to control and monitor physical variables and undertake data treatment. Researchers could use centralized automation in their projects to facilitate the collection of a large amount of data. This could help them to not only understand the building envelope behavior, but also develop new services to be integrated into the market of BASs

Currently in BASs, there is no evidence of integration of in-use building energy monitoring systems to characterize the TEP. However, it would be useful to know how efficient the envelope is after the construction or retrofit in order to determine the discrepancy between the building’s design and the building in-use, and to identify future retrofits of the building envelope.

The equipment necessary to carry out the TEP characterization includes sensors, controllers, software, hardware, communication protocols, and other devices and components of MCSs. At the end of the 1970s and the beginning of the 1980s, studies were undertaken regarding the different monitoring technologies and cost/precision criteria for equipment selection used in the energy monitoring of buildings to characterize the TEP of buildings with the estimation of HLC together with other estimations. Currently, there is no evidence from recent studies comparing between the different sensors and equipment used in energy monitoring with existing technologies. There is also no evidence for which monitoring systems should be used to characterize the TEP in BASs or domotic systems.

The reviewed publications do not specify the selection criteria of the monitoring systems used in research projects, which shows that there is no standardization in the type of MCS that should be used to perform experimental tests in these estimations. It is also evident that experimental tests tend to focus more on developing methods to estimate the HLC and other estimations to determinate the envelope energy behaviors of buildings to characterize the TEP, rather than carrying out an analysis to determine the criteria to choose the MCSs. This trend is apparent even though the sensors used to measure physical variables are critical to the reliability of the data collected to perform the TEP characterization. It has been observed, too, that the MCSs used to estimate HLC allow the analysis and estimation of other parameters used to characterize the buildings’ TEP. The physical variables necessary for these estimations are collected in current BASs and domotic systems in order to determine user comfort, electricity consumption, and for the control of the building systems. This makes it possible for the experimental tests used to characterize TEP to be designed from the perspective of BASs and domotic systems, in order to introduce this characterization into these automation systems. For this to be effective, the experimental tests should develop selection criteria for the MCSs in the research projects in order to standardize them.

The standardization of the MCSs used in the TEP characterization in experimental tests needs further research in order to ensure the physical data are accurate enough to rigorously apply the HLC estimation methods. In this way, the HLC estimates for the emission of reliable EPCs according to the requirements of the legislation may be used if they are able to be integrated into BASs and domotic systems. It is also necessary to emphasize the importance of defining the criteria in MCS selection in order to guarantee the technologies are accurate, reliable, profitable, and safe from cyber-attacks.

No publication has been found that develops AFDD methods for the whole monitoring system of a building in BASs or domotic systems. Studies that characterize TEP by testing different sensor technologies to understand any discrepancies in the HLC estimation, and what the sensor response is in building energy monitoring systems, are also lacking. The methods analyzed to estimate the HLC and other estimates to determinate the envelope energy behaviors of building taking into account the errors and the manufacturing precision of the devices used. As an example, to understand the measured discrepancies of temperature, RH, CO_2_ levels, energy consumption, solar radiation, and other physical variables, it is necessary to know the sensor characteristics used in a building’s automation in the research projects that characterize the TEP. For this, it is essential to know in real-time the faults that occur during the experimental tests, in order to analyze their impact and determine the error discrepancies with the manufacturing data sheet. All of this information is necessary for the implementation of AFDD method in the MCSs of experimental tests.

The literature studied in this paper evidences the use of AFDD methods in building systems like fan coils, HVACs, heat pumps, air conditioners, commercial refrigerators, lighting, water heaters, chillers, cooling towers, AHUs and VAV boxes. However, a specific method for all MCSs used in BASs and domotic systems has not been found, with this being essential in order to integrate FDD methods for all parties that make up these MCSs. It is necessary to develop FDD methods to calibrate, predict and detect the error of all devices in an MCS. This would facilitate the maintenance of the system, allowing its self-regulation and calibration to increase the accuracy and reliability of the studies.

In future, research needs to focus on the effect of the estimation of the HLC and other estimations to determinate the envelope energy behaviors of buildings using different sensor technologies, with laboratory accuracy and market sensor accuracy. This type of research could allow the development of a monitoring kit and control specifications to characterize the TEP, together with their layout in buildings, in order to in the future issue reliable certificates of the energy performance of building with the EPCs. In addition, it is necessary to know the discrepancy in the estimations of the HLC and other estimations to determinate the envelope energy behaviors of buildings. This discrepancy can be determined using the technology of current BASs and domotic systems in order to know if, with the market technology, it is possible to determine the TEP of buildings after the new buildings construction or retrofit of existing buildings. Therefore, knowing how to integrate the standardized MCSs used to estimate the HLC and other estimations to determinate the envelope energy behaviors of buildings, which characterize the TEP, in BASs and domotic systems for new and existing buildings, is essential.

## Figures and Tables

**Table 1:** Differences between the wired and wireless communication protocols [30].

Wired	Wireless
High bandwidth	Low-medium bandwidth
High performance	Higher latency
Robust	Interference
Reliable	Unreliable by nature
Installation expensive	Installation cheap
“Unlimited” resources	Low power, memory
Static network	Mobile network
Less security problems	More security problems

**Table 2:** The main sensors and meters used for control in building automation based on previous research [17,29].

Typology	Sensor Measure	International System Unit
Total Consumption	Electricity of whole Buildings	Wh, kWh, MWh
	Energy Consumption of Heating, Cooling, light, etc.	Wh, kWh, MWh
	Water Consumption	L, m^3^
	Fuel Consumption	Wh, kWh, MWh, L, Nm^3^, m^3^
Weather	Temperature	°C
	Relative humidity	%
	Global Solar Radiation	W/m^2^
	Wind Velocity	km/h
	Wind Direction	(0°–360°)
Indoor Conditions	Temperature	°C
	Relative Humidity	%
	CO_2_ Concentration	ppm
	Illuminance Level (Lux)	lux
Building Systems	Fluid Temperature of Circuit: AHU/HVAC and Hot Water	°C
	AHU/HVAC Relative Humidity	%
	Flows	L/h, m^3^/s
	Pressures	kPa, Pa
	Presence Sensor’s Control	0–100%, 0–1, ON/OFF, 0/1
	CO_2_ Sensor’s Control	0–100%, 0–1, ON/OFF, 0/1
Frequency to Collect Data	High, Medium and Low Frequency	s, min, h, day, day, month, year

**Table 3:** Examples of impacts produced by sensor errors in some study cases.

Reference	Error and Fault Analyzed	Impact
R. Zhang, T. Hong [55]	Outdoor air temperature sensor errors and thermostat errors on energy consumption.	Increase of cooling energy consumption by 0.8–13.6%, cooling and heating energy consumption increases 19.07–34.24%.
J. Verhelst, G. V. Ham [56]	HVAC performance under the fault sensors and actuators in a concrete core activated office building.	Economic impact from +7% to +1000% due to simultaneous sensor and actuator faults (realistic, randomly distributed and non-correlative).
K. Roth, D. Westphalen [57]	Identify thirteen key faults based on literature review, developing bottom-up energy impact range.	Increase of 4–18% of the energy annual consumption of the sum of commercial building HVAC, lighting, and refrigeration energy consumption, and is consistent with the typical range of energy waste reported in building commissioning studies.
J.Y. Kao, E.T. Pierce [58]	Simulation of error effects in the sensors of automatic controls for HVAC systems, in an office building of lightweight construction.	In annual building-energy requirements, increase of 30–50% attributable to an air handling system.
W. Kim [59]	Fault detection and diagnosis for air conditioners and heat pumps based on virtual sensors.	Reduction of approximately 20% of the cooling capacity and 15% of the energy efficiency if the refrigerant undercharging is in the range of 25%.

**Table 4:** Physical variables measured in the Corrected Average Method.

Typology	Sensor Measure	International System Unit
Energy Consumption	Total electricity consumed whiting the buildings envelope	Wh, kWh, MWh
	Total energy supplied by the Heating	Wh, kWh, MWh
Weather	Outdoor temperature	°C
	Horizontal global solar radiation	W/m^2^
Indoor Conditions	Indoor temperature	°C

**Table 5:** Physical variables measured in the Co-Heating test.

Typology	Sensor Measure	International System Unit
Energy Consumption	Total electricity within the building’s envelope	Wh, kWh, MWh
	Total energy consumption by the heaters and fans	Wh, kWh, MWh
Weather	Outdoor temperature	°C
	Vertical global south solar radiation	W/m^2^
Indoor Conditions	Indoor temperature	°C

**Table 6:** Monitoring system of a public building of the University of the Basque Country.

Typology	Measurement	Device Identification	Accuracy
Energy consumption	Heating system	7 Calorimeter: Kamstrup Multical 602 for heating; F0 1 calorimeter; F1, F2 and F3 2 calorimeters per floor, for the set sensors	E_T_ ± (0.4 + 4/ΔT)%
	Lighting system	4 Electricity Power Meter: 1 ABB EM/S 3.16.1 meter, 3 ABB A43 meters (1 per floor)	±2% for all
Indoor Conditions	Illuminance (lux)	13 Illuminance sensors: Siemens 5WG1 255-4AB12	-
	Air Quality (ppm CO_2_)	13 Air quality, Temperature and Humidity Sensors: ARCUS SK04-S8-CO2-TF	±1% Measurement Error
	Temperature (°C)		±0.5 °C
	Relative Humidity (%)		±3% RH
Weather	Illuminance (lux)	1 Weather Station on roof: ELSNER 3595 Sun tracer KNX basic	±35% at 0…150,000 lux
	Temperature (°C)		±0.5 °C
	Wind Speed (m/s)		±25% at 0…15 m/s
	Rain (yes/no)		-
	Temperature (°C)	1 Outdoors Temperature and Humidity Sensor on roof: ARCUS SK01-TFK-AFF	±0.5 °C
	Relative Humidity (%)		±3% RH
	Global Horizontal Solar Radiation (W/m^2^)	1 Pyranometer on roof: ARCUS SK08-GLBS	±5%

**Table 7:** Controlling system of a public building of the University of the Basque Country.

Typology	Technology	Device Specifications	Descriptions
Communications	KNX Protocol	Bus KNX	The installation is based on device communication via a communication bus KNX that will allow communication between all the devices present in the installation.
	Cable	Twisted pair (TP1) of the type Y (St) Y 2 × 2 × 0.8 mm^2^	Red (+) and black (–) for the bus line. The two remaining wires are yellow and white, which will be used for additional applications, additional power supply of certain components, or as an additional bus line or reserve for breakdowns.
Hardware	KNX/IP Interface	Weinzierl 730	Four lines of the Measuring System and of the lines set out are done through IP connections. Each line has a KNX/IP Interface located on the KNX board of each floor.
	Web Server	For the control and monitoring of the installation, the Cambridge Studio Evolution Server (CBSE) of IPAS is used	This device must be connected to a LAN network of each building and provided with Internet access. It communicates with the KNX network using KNX/IP gateways.
	Switch and router	Used by university	The university has several routers and switches that were used.
Software	Specific KNX software tool	Unique Standard Application for Programming KNX Systems Software.	The programming occurs in two different phases. The first phase is the creation of the topological structure of the installation, parameterization of the devices, and assigning of the physical addresses and groups. The second phase consists of the physical programming of the installation directly into the building.

**Table 8:** List of publications used in the MCS to characterize the TEP of in-situ buildings through HLC estimation and other estimates used to determinate energy behavior of the buildings.

Reference	Publication Year	TEP CHARACTERIZATION THROUGH									
		HLC Estimation				Estimation of Building Envelope Energy Behavior Through					
		Co-Heating Method	Regression Method	Average Method	Corrected Average Method	Energy Consumption	Energy Balance Estimation	Infiltration Estimation	*U*-Value Estimation	*R*-Value Estimation	Others Estimation and Methods ^1^
[70]	1978	X				X		X			
[73]	1979	X									
[74]	1979	X									X
[75]	1980	X						X			
[76]	1985		X				X	X			X
[77]	1985		X				X				X
[78]	1995										X
[79]	2000	X				X					
[80]	2001					X					
[81]	2005	X									
[82]	2007	X									
[83]	2013	X						X	X		
[84]	2015	X									
[85]	2015										X
[86]	2015	X						X			
[87]	2015					X	X				X
[88]	2016	X						X	X		
[17]	2016		X		X						
[89]	2016	X							X		
[90]	2017	X									
[91]	2017	X							X	X	
[92]	2017		X	X							X
[93]	2018	X									
[72]	2018	X									

**^1^** Includes others estimations of building energy behavior as the estimations of the heat dynamics of buildings, thermal performance evaluations, and so forth, using other methods, are presented in Table 8. The methods used are statistical methods and other models of calculus (e.g., Grey Box Modelling, multiple linear regression, ARX and ARMAX models, etc.).

**Table 9:** MCSs in the reviewed literature from 1979–2018, specified by the authors to characterize the TEP of buildings.

Reference	Publication Year	Type of Publication	FDD	Sensors																							Actuators		Control System						Other Devices			
			Specify the Application of FDD Method to MCS	Indoor Air Temperature	Surface Temperature (Out and Indoor)	Indoor CO_2_	Interior Relative Humidity	Heat Fluxes	Infiltration	Infrared Thermography	Illuminance Level (Lux)	Total Electricity Meter	Gas Meter	Heat Meter	HVAC Air Flow	Light Electricity Meter	Outdoor Air Temperature	Exterior Relative Humidity	Global Vertical Solar Radiation Intensity	Global Horizontal Solar Radiation Intensity	Diffuse Solar Radiation Intensity	Outdoor Illuminance Level (Lux)	Wind Speed Anemometer	Wind Direction	Atmospheric Pressure	Precipitation	Thermostat	Other Building Devices to Control	Protocol Communication	Getaway or Transmitters	Data Logger	Data Processor	SCADA	Computer	Building Heating Systems	HVAC	Fans	Dedicated Electric Radiator
[70]	1978	Report	No	X					X	X		X	X	X		X	X	X	X	X	X		X	X		X	X	X	X		X	X		X				
[73]	1979	Paper	No	X					X			X					X										X								X			
[74]	1979	Report	No	X								X					X		X	X								X			X			X				
[75]	1980	Report	No	X					X			X															X				X	X			X			X
[76]	1985	Report	No	X	X	X		X	X			X	X	X				X	X	X	X		X	X			X	X			X	X		X				
[77]	1985	Report	No	X								X	X	X			X		X	X			X	X							X			X				
[78]	1995	Paper	No	X					X								X		X									X										X
[79]	2000	Paper	No	X								X																			X	X						
[80]	2001	Paper	No	X					X			X					X		X										X			X						
[81]	2005	Paper	No	X			X					X					X	X		X							X								X			
[82]	2007	Paper	No	X						X		X					X	X	X								X		X	X	X			X			X	X
[83]	2013	Paper	No	X				X	X	X		X					X																				X	X
[84]	2015	Conference Paper	No	X								X					X		X										X		X	X		X			X	X
[85]	2015	Conference Paper	No	X													X	X	X	X	X		X	X				X			X			X	X	X		
[86]	2015	Conference Paper	No	X					X								X		X	X	X		X	X														
[87]	2015	Paper	No	X		X	X				X	X			X		X		X	X	X										X	X				X		
[88]	2016	Paper	No	X	X		X	X	X			X					X		X	X	X																	X
[17]	2016	Paper	No	X		X	X				X			X		X	X	X		X		X	X															
[89]	2016	Paper	No	X				X				X		X			X	X	X				X	X			X	X			X				X		X	
[90]	2017	Paper	No	X				X	X			X			X		X												X		X	X				X	X	X
[91]	2017	Paper	No	X				X	X	X		X					X							X			X				X					X	X	X
[92]	2017	Paper	No	X	X			X				X		X			X	X	X	X	X		X	X														
[93]	2018	Paper	No	X				X				X	X				X										X									X	X	X
[72]	2018	Paper	No	X					X	X		X							X	X	X						X										X	X

**Table 10:** Description of the level quantification used to analyze the MCSs presented in the reviewed literature.

Levels	Detail Degree of Technical Specifications	Quantitative Value
Level A	High degree specification	1
Level B	Partial specification	0.5
Level C	There is not specification	0

**Table 11:** Criteria to evaluate the specification level.

ANALIZED CRITERIALS	
Devices of Monitoring System	Devices of Controlling System and Data Acquisition System
Specify the Model or Type	Specify the Model or Type of control devices
Specify the Data Sheet	Specify the Data Sheet
Details the Accuracy	Specify the Protocol Communications
Specify the criterion used for determinate the Type of Monitoring System	Specify the operating characteristics of Hardware and Software
	Specify the Hardware and Software type
	Specify the criterion used for determinate the type of Controlling System used

**Table 12:** Qualitative analysis of the specification level’s degree of the analyzed criteria for the devices, hardware, and software of MCSs used in buildings or prototypes in each publication studied.

Reference	Publication Year	Type of Publication	Monitoring System’s Devices				Controlling System’s Devices					
			Specify the Model or Type	Specify the Data Sheet	Details the Accuracy	Specify the Criterion used for Determinate the Type of Monitoring System	Specify the Model or Type of control Devices	Specify the Data Sheet	Specify the Protocol Communications	Specify the Operating Characteristics of Hardware and Software	Specify the Hardware and Software Type	Specify the Criterion Used for Determinate the Type of Controlling System Used
[70]	1978	Report	Level C	Level C	Level C	Level C	Level C	Level B	Level A	Level A	Level B	Level C
[73]	1979	Paper	Level C	Level C	Level C	Level C	Level C	Level C	Level C	Level C	Level C	Level C
[74]	1979	Report	Level A	Level A	Level A	Level A	Level A	Level A	Level A	Level A	Level A	Level A
[75]	1980	Report	Level C	Level C	Level C	Level C	Level C	Level C	Level C	Level A	Level C	Level C
[76]	1985	Report	Level B	Level B	Level B	Level B	Level B	Level B	Level C	Level A	Level A	Level A
[77]	1985	Report	Level B	Level B	Level B	Level B	Level C	Level C	Level C	Level A	Level A	Level A
[78]	1995	Paper	Level C	Level C	Level C	Level C	Level C	Level B	Level C	Level C	Level C	Level B
[79]	2000	Paper	Level C	Level C	Level C	Level C	Level C	Level C	Level C	Level C	Level B	Level C
[80]	2001	Paper	Level C	Level C	Level C	Level C	Level C	Level C	Level B	Level B	Level B	Level C
[81]	2005	Paper	Level B	Level C	Level C	Level C	Level A	Level A	Level C	Level C	Level C	Level B
[82]	2007	Paper	Level A	Level C	Level C	Level C	Level A	Level A	Level A	Level A	Level A	Level C
[83]	2013	Paper	Level C	Level C	Level C	Level C	Level C	Level C	Level C	Level C	Level C	Level C
[84]	2015	Conference Paper	Level C	Level C	Level C	Level C	Level C	Level C	Level A	Level B	Level B	Level B
[85]	2015	Conference Paper	Level C	Level C	Level C	Level C	Level C	Level C	Level C	Level C	Level B	Level B
[86]	2015	Conference Paper	Level C	Level C	Level C	Level C	Level C	Level C	Level C	Level C	Level C	Level C
[87]	2015	Paper	Level C	Level C	Level C	Level C	Level C	Level C	Level C	Level C	Level A	Level C
[88]	2016	Paper	Level B	Level C	Level B	Level C	Level C	Level C	Level C	Level C	Level C	Level C
[17]	2016	Paper	Level A	Level C	Level A	Level B	Level C	Level C	Level C	Level C	Level C	Level C
[89]	2016	Paper	Level A	Level C	Level A	Level C	Level C	Level B	Level C	Level B	Level B	Level C
[90]	2017	Paper	Level A	Level C	Level B	Level C	Level C	Level C	Level A	Level B	Level B	Level C
[91]	2017	Paper	Level A	Level C	Level A	Level B	Level C	Level B	Level C	Level B	Level B	Level C
[92]	2017	Paper	Level A	Level C	Level C	Level C	Level C	Level C	Level C	Level C	Level C	Level C
[93]	2018	Paper	Level C	Level B	Level B	Level C	Level C	Level B	Level C	Level C	Level C	Level C
[72]	2018	Paper	Level A	Level C	Level B	Level C	Level C	Level B	Level C	Level C	Level C	Level C

**Table 13:** Quantitative analysis of specification level’s degree of the analyzed criteria of MCSs used in buildings or prototypes in the reviewed literature.

**All Reviewed Literature**										
**All Methods**	**Monitoring System’s Devices**				**Controlling System’s Devices**					
**24 references**	Specify the Model or Type	Specify the Data Sheet	Details the Accuracy	Specify the criterion used for determinate the Type of Monitoring System	Specify the Model or Type of control devices	Specify the Data Sheet	Specify the Protocol Communications	Specify the operating characteristics of Hardware and Software	Specify the Hardware and Software type	Specify the criterion used for determinate the type of Controlling System used
**Level A**	8	1	4	1	3	3	5	6	5	3
	33.3%	4.2%	16.7%	4.2%	12.5%	12.5%	20.8%	25%	20.8%	12.5%
**Level B**	4	3	6	4	1	7	1	5	8	4
	16.7%	12.5%	25%	16.7%	4.2%	29.2%	4.2%	20.8%	33.3%	16.7%
**Level C**	12	20	14	19	20	14	18	13	11	17
	50%	83.3%	58.3%	79.2%	83.3%	58.3%	75.0%	54.2%	45.8%	70.8%
**Reviewed Literature with Co-Heating Method (HLC estimation)**										
**Co-Heating Method**	**Monitoring System’s Devices**				**Controlling System’s Devices**					
**16 references** **67%**	Specify the Model or Type	Specify the Data Sheet	Details the Accuracy	Specify the criterion used for determinate the Type of Monitoring System	Specify the Model or Type of control devices	Specify the Data Sheet	Specify the Protocol Communications	Specify the operating characteristics of Hardware and Software	Specify the Hardware and Software type	Specify the criterion used for determinate the type of Controlling System used
**Level A**	6	1	3	1	3	3	5	4	2	1
	37.5%	6.3%	18.8%	6.3%	18.8%	18.8%	31.3%	25%	12.5%	6.3%
**Level B**	2	1	4	1	0	5	0	4	6	2
	12.5%	6.3%	25%	6.3%	0%	31.3%	0%	25%	37.5%	12.5%
**Level C**	8	14	9	14	13	8	11	8	8	13
	50%	87.5%	56.3%	87.5%	81.3%	50%	68.8%	50%	50%	81.3%
**Reviewed Literature with Regression Method (HLC estimation)**										
**Regression Methods**	**Monitoring System’s Devices**				**Controlling System’s Devices**					
**4 references** **17%**	Specify the Model or Type	Specify the Data Sheet	Details the Accuracy	Specify the criterion used for determinate the Type of Monitoring System	Specify the Model or Type of control devices	Specify the Data Sheet	Specify the Protocol Communications	Specify the operating characteristics of Hardware and Software	Specify the Hardware and Software type	Specify the criterion used for determinate the type of Controlling System used
**Level A**	2	0	1	0	1	0	0	0	2	2
	50%	0%	25%	0%	25%	0%	0%	0%	50%	50%
**Level B**	2	2	2	3	0	1	1	0	0	0
	50%	50%	50%	75%	0%	25%	25%	0%	0%	0%
**Level C**	0	2	1	1	0	3	3	4	2	2
	0%	50%	25%	25%	0%	75%	75%	100%	50%	50%
**Reviewed Literature with Average Method (HLC estimation)**										
**Average Method**	**Monitoring System’s Devices**				**Controlling System’s Devices**					
**1 reference** **4%**	Specify the Model or Type	Specify the Data Sheet	Details the Accuracy	Specify the criterion used for determinate the Type of Monitoring System	Specify the Model or Type of control devices	Specify the Data Sheet	Specify the Protocol Communications	Specify the operating characteristics of Hardware and Software	Specify the Hardware and Software type	Specify the criterion used for determinate the type of Controlling System used
**Level A**	1	0	0	0	0	0	0	0	0	0
	100%	0%	0%	0%	0%	0%	0%	0%	0%	0%
**Level B**	0	0	0	0	0	0	0	0	0	0
	0%	0%	0%	0%	0%	0%	0%	0%	0%	0%
**Level C**	0	1	1	1	1	1	1	1	1	1
	0%	100%	100%	100%	100%	100%	100%	100%	100%	100%
**Reviewed Literature with Corrected Average Method (HLC estimation)**										
**Corrected Average Method**	**Monitoring System’s Devices**				**Controlling System’s Devices**					
**1 reference** **4%**	Specify the Model or Type	Specify the Data Sheet	Details the Accuracy	Specify the criterion used for determinate the Type of Monitoring System	Specify the Model or Type of control devices	Specify the Data Sheet	Specify the Protocol Communications	Specify the operating characteristics of Hardware and Software	Specify the Hardware and Software type	Specify the criterion used for determinate the type of Controlling System used
**Level A**	1	0	1	0	0	0	0	0	0	0
	100%	0%	100%	0%	0%	0%	0%	0%	0%	0%
**Level B**	0	0	0	1	0	0	0	0	0	0
	0%	0%	0%	100%	0%	0%	0%	0%	0%	0%
**Level C**	0	1	0	0	1	1	1	1	1	1
	0%	100%	0%	0%	100%	100%	100%	100%	100%	100%
**Reviewed Literature implementing other methods**										
**Other Methods Applied ^1^**	**Monitoring System’s Devices**				**Controlling System’s Devices**					
**7 references** **29%**	Specify the Model or Type	Specify the Data Sheet	Details the Accuracy	Specify the criterion used for determinate the Type of Monitoring System	Specify the Model or Type of control devices	Specify the Data Sheet	Specify the Protocol Communications	Specify the operating characteristics of Hardware and Software	Specify the Hardware and Software type	Specify the criterion used for determinate the type of Controlling System used
**Level A**	1	0	0	0	0	0	0	2	3	2
	14%	0%	0%	0%	0%	0%	0%	29%	43%	29%
**Level B**	2	2	2	2	1	2	1	1	2	2
	29%	29%	29%	29%	14%	29%	14%	14%	29%	29%
**Level C**	4	5	5	5	6	5	6	4	2	3
	57%	71%	71%	71%	86%	71%	86%	57%	29%	43%

**^1^** This quantitative analysis include experimental test that use other methods: (i) To estimate HLC using methods different to Co-Heating Method, Average Method and Corrected Average Method. (ii) To characterize the Thermal Envelope performance (TEP).

**Table 14:** Quantitative analysis of the references studied by methodology: Global analysis of the method used, the fault detection and sensor type used for measuring physical variables in the MCSs in each methodology.

Literatures Grouping by Methods	Type of Publication	Global Analysis	FDD	Sensors																						
			Specify the Application of Fault Detection Method	Indoor Air Temperature	Surface Temperature (Out and Indoor)	Indoor CO_2_	Interior Relative Humidity	Heat Flow	Infiltration	Infrared Thermography	Indoor Illumination Level (Lux)	Total Electricity Meter	Gas Meter	Heat Meter	HVAC Air Flow	Light Electricity Meter	Outdoor Air Temperature	Exterior Relative Humidity	Global Vertical Solar Radiation Intensity	Global Horizontal Solar Radiation Intensity	Diffuse Solar Radiation Intensity	Outdoor Illuminance Level (Lux)	Wind Speed Anemometer	Wind Direction	Atmospheric Pressure	Precipitation
**All Literatures Studied**	Total references	**24**	0	24	3	3	4	8	12	5	2	20	4	6	2	2	20	8	15	12	8	1	8	8	0	1
	Percentage rate	**100%**	0%	100%	13%	13%	17%	33%	50%	21%	8%	83%	17%	25%	8%	8%	83%	33%	63%	50%	33%	4%	33%	33%	0%	4%
**Co-Heating Method**	Total references	**16**	0	16	1	0	2	6	9	5	0	15	2	2	1	1	13	4	8	6	4	0	3	4	0	1
	Percentage rate	**67%**	0%	100%	6%	0%	13%	38%	56%	31%	0%	94%	13%	13%	6%	6%	81%	25%	50%	38%	25%	0%	19%	25%	0%	6%
**Regression Method**	Total references	**4**	0	4	2	2	1	2	1	0	1	3	2	4	0	1	3	3	3	4	2	1	4	3	0	0
	Percentage rate	**17%**	0%	100%	50%	50%	25%	50%	25%	0%	25%	75%	50%	100%	0%	25%	75%	75%	75%	100%	50%	25%	100%	75%	0%	0%
**Average Method**	Total references	**1**	0	1	1	0	0	1	0	0	0	1	0	1	0	0	1	1	1	1	1	0	1	1	0	0
	Percentage rate	**4%**	0%	100%	100%	0%	0%	100%	0%	0%	0%	100%	0%	100%	0%	0%	100%	100%	100%	100%	100%	0%	100%	100%	0%	0%
**Corrected Average Method**	Total references	**1**	0	1	0	1	1	0	0	0	1	0	0	1	0	1	1	1	0	1	0	1	1	0	0	0
	Percentage rate	**4%**	0%	100%	0%	100%	100%	0%	0%	0%	100%	0%	0%	100%	0%	100%	100%	100%	0%	100%	0%	100%	100%	0%	0%	0%
**Other Methods Applied**	Total references	**7**	0	7	2	2	1	2	3	0	1	5	2	3	1	0	6	3	7	5	4	0	4	4	0	0
	Percentage rate	**29%**	0%	100%	29%	29%	14%	29%	43%	0%	14%	71%	29%	43%	14%	0%	86%	43%	100%	71%	57%	0%	57%	57%	0%	0%

**Table 15:** Quantitative analysis of references studied by methodology: Global analysis of the method, actuators, controls systems, and devices used in each methodology.

Literatures Grouping by Methods	Type of Publication	Global Analysis	Actuators		Control System						Other Devices			
			Thermostat	Other Building Devices to Control	Protocol Communication	Getaway or Transmitters	Data Logger	Data Processor	SCADA	Computer	Building Heating Systems	HVAC	Fans	Dedicated Electric Radiator
**All Literatures Studied**	Total references	**24**	10	6	5	1	13	8	0	7	5	5	8	10
	Percentage rate	**100%**	42%	25%	21%	4%	54%	33%	0%	29%	21%	21%	33%	42%
**Co-Heating Method**	Total references	**16**	9	3	4	1	9	5	0	4	4	3	8	9
	Percentage rate	**67%**	56%	19%	25%	6%	56%	31%	0%	25%	25%	19%	50%	56%
**Regression Method**	Total references	**4**	1	1	0	0	2	1	0	2	0	0	0	0
	Percentage rate	**17%**	25%	25%	0%	0%	50%	25%	0%	50%	0%	0%	0%	0%
**Average Method**	Total references	**1**	0	0	0	0	0	0	0	0	0	0	0	0
	Percentage rate	**4%**	0%	0%	0%	0%	0%	0%	0%	0%	0%	0%	0%	0%
**Corrected Average Method**	Total references	**1**	0	0	0	0	0	0	0	0	0	0	0	0
	Percentage rate	**4%**	0%	0%	0%	0%	0%	0%	0%	0%	0%	0%	0%	0%
**Other Methods Applied**	Total references	**7**	1	3	1	0	4	3	0	3	1	2	0	1
	Percentage rate	**29%**	14%	43%	14%	0%	57%	43%	0%	43%	14%	29%	0%	14%

## Appendix A

**Table A1:** Acronyms list.

Acronym	Meaning
A2PBEER	Affordable and Adaptable Public Buildings through Energy Efficient Retrofitting
AFDD	Automated Fault Detection and Diagnosis
AHU	Air Handling Unit
ANSI	American National Standards Institute
AR	Autoregressive
ARMA	Autoregressive Moving Average
ANN	Artificial Neural Network
ASHRAE	American Society of Heating, Refrigeration and Air Conditioning Engineers
BACnet	Data Communication Protocol for Building Automation and Control Networks
BAS	Building Automation Systems
BMS	Building Management System
CO_2_	Carbon Dioxide
CSIC	Superior Council of Scientific Investigations of Spain
EED	Energy Efficiency Directive
EPBD	Energy Performance of Buildings Directive
EPCs	Energy Performance Certificates
EU	Europe Union
FDD	Fault Detection and Diagnosis
FP7	7th Framework Programme for Research and Technological Development
HLC	Heat Loss Coefficient
HTC	Heat Transfer Coefficient
HVAC	Heating, Ventilation and Air Conditioning systems
IEA	International Energy Agency
IEEE	Institute of Electrical and Electronics Engineers
IoT	Internet of Things
KPI	Key Performance Indicators
LONWork	Local Operating Network
MCS	Monitoring and Controlling System
Mtoe	Million Tons of Oil Equivalent
OSGI	Open Services Gateway Initiative
OSI	Open System Interconnection
PCA	Principal Component Analysis
PLC	Programmable Logic Controllers
PCA	Principal Component Analysis
RD&D	Research, Development and Design
RF	Radio Frequency
RH	Relative Humidity
SAP	Standard Assessment Procedure
SCADA	Supervisory Control And Data Acquisition
SVM	Support Vector Machine
TCP/IP	Transmission Control Protocol / Internet Protocol
TEP	Thermal Envelope Performance
UK	United Kingdom
UPnP	Universal Plug and Play
VAV	Variable Air Volume
WPAN	Wireless Personal Area Networks
